# Diffusion-tensor magnetic resonance imaging as a non-invasive assessment of extracellular matrix remodeling in lumbar paravertebral muscles of rats with sarcopenia

**DOI:** 10.1186/s12891-024-07654-0

**Published:** 2024-07-13

**Authors:** Xin-Chen Huang, Ji-Yao Ma, Chao Gao, Jia-Xin Chen, Chun-Li Li, Yi-Long Huang, Bo He

**Affiliations:** 1 Department of Radiology, The First Affiliated Hospital of Kunming Medical University, Kunming, Yunnan, China; 2 Department of Radiology, The Second Affiliated Hospital of Zhejiang University School of Medicine, Hangzhou, China

**Keywords:** Diffusion-tensor magnetic resonance imaging, Extracellular matrix remodeling, Animal model, Sarcopenia, Lumbar paravertebral muscles

## Abstract

**Background:**

Extracellular matrix (ECM) remodeling in skeletal muscle is a significant factor in the development of sarcopenia. This study aims to evaluate changes in ECM remodeling in the lumbar paravertebral muscles of sarcopenic rats using diffusion-tensor magnetic resonance imaging (DT-MRI) and compare them with histology.

**Methods:**

Twenty 6-month-old female Sprague Dawley rats were randomly divided into the dexamethasone (DEX) group and the control (CON) group. Both groups underwent 3.0T MRI scanning, including Mensa, T2WI, and DT-MRI sequences. The changes in muscle fibers and extracellular matrix (ECM) of the erector spinal muscle were observed using hematoxylineosin and sirius red staining. The expressions of collagen I, III, and fibronectin in the erector spinae were detected by western blot. Pearson correlation analysis was employed to assess the correlation between MRI quantitative parameters and corresponding histopathology markers.

**Results:**

The cross-sectional area and fractional anisotropy values of the erector spinae in the DEX group rats were significantly lower than those in the CON group (*p* < 0.05). Hematoxylin eosin staining revealed muscle fiber atrophy and disordered arrangement in the DEX group, while sirius red staining showed a significant increase in collagen volume fraction in the DEX group. The western blot results indicate a significant increase in the expression of collagen I, collagen III, and fibronectin in the DEX group (*p* < 0.001 for all). Correlation coefficients between fractional anisotropy values and collagen volume fraction, collagen I, collagen III, and fibronectin were − 0.71, -0.94, -0.85, and − 0.88, respectively (*p* < 0.05 for all).

**Conclusions:**

The fractional anisotropy value is strongly correlated with the pathological collagen volume fraction, collagen I, collagen III, and fibronectin. This indicates that DT-MRI can non-invasively evaluate the changes in extracellular matrix remodeling in the erector spinal muscle of sarcopenia. It provides a potential imaging biomarker for the diagnosis of sarcopenia.

**Supplementary Information:**

The online version contains supplementary material available at 10.1186/s12891-024-07654-0.

## Introduction

Sarcopenia is a progressive, generalized syndrome characterized by muscle weakness, loss of muscle mass, and rapid decline in muscle function. It is associated with an increased incidence of adverse outcomes, such as extrapulmonary conditions [1], chronic liver disease [2], fall fractures, and death [3]. With the intensification of global population aging and the extension of life expectancy, it is predicted that the number of patients with sarcopenia will reach 500 million by 2050, placing a significant burden on the healthcare system and society [4]. The development of sarcopenia is widely believed to have a multifactorial etiology, including inflammatory damage, sparse capillarization, mitochondrial dysfunction, decreased satellite cell count, and hormonal imbalance, etc [5]. Recent studies have shown that with age, connective tissue and, in particularly, extracellular matrix (ECM) proteins accumulate in skeletal muscle, resulting in a stiff microenvironment [6]. The characteristic pathological changes of skeletal muscle ECM remodeling mainly include collagen deposition and skeletal muscle fibrosis [7, 8]. Abnormal ECM remodeling has been shown to affect the functional properties of skeletal muscle, with further consequences for the role of transmission of tension between adjacent myocytes, and is an important reason of reduced muscle strength [9]. Because these tissues lack the ability to contract, it disturbs the nutrient supply to muscle fibers and increases the susceptibility of muscles to reinjury, which in turn impairs muscle function [10]. The evaluation of the ECM is gaining increasing attention due to its role in regulating skeletal muscle function and adaptation [11]. Histopathology can detect these features of muscles. However, histology assessment is invasive and semi-quantitative, and can only detect local changes in the muscle, unable to evaluate the overall quality and composition of skeletal muscle. Therefore, developing an alternative method to accurately monitor the pathological progression of skeletal muscle fibrosis is crucial.

Magnetic resonance imaging (MRI) is considered the gold standard for non-invasive quantitative assessment of muscle quantity and quality [12]. Because of its non-invasive nature, superior soft tissue resolution, visualization of markers, and reliability of measurements [13]. Wherein, the diffusion-tensor magnetic resonance imaging (DT-MRI) technique has been proposed as an effective method for evaluating the ECM content of macromolecules [14]. DT-MRI has been used to distinguish between different degrees of fibrosis in chronic hepatitis [15], to predict early renal fibrosis in patients with kidney disease [16], and to evaluate diffuse myocardial fibrosis in patients with chronic end-stage heart failure [17]. However, there have been few studies evaluating the value of DT-MRI in skeletal muscle ECM remodeling in sarcopenia. Although evaluating pathological changes in clinical patients to validate imaging results is more reliable, invasive muscle biopsy is often not easily accepted by patients. Therefore, this study established a classic dexamethasone (DEX)-induced sarcopenic rat model.

Thus, our study aims to non-invasively monitor the imaging changes of the lumbar paravertebral muscles in sarcopenic rats using DT-MRI technology and to evaluate its correlation with histological parameters. This analysis may identify DT-MRI as an imaging biomarker for sarcopenia that can capture muscle fiber changes related to ECM remodeling without the need for biopsies.

## Method

### Animals

All rats were purchased from Kunming Medical University Animal Experiment Center [SYXK (Yunnan) K2020-0006], all animal experiments had been approved by the Institutional Animal Care and Use Committee of Kunming Medical University (approval number: kmmu20221517). The sample size was previously evaluated by power analysis and adhered to the 3R principles (reduction, replacement and refinement) of the China Council of Animal Care’s Ethic of Animal Experimentation [18].

The study included twenty female Sprague-Dawley rats aged 6 months and weighing 300 ± 50 g. The rats were fed standard rat chow, with diets refreshed daily. They were randomly divided into two groups: a DEX-induced sarcopenia group and a control (CON) group. The CON group (*n* = 10) was administrated with saline. Referring to the modelling methods of Fappi et al. [19]. the DEX group (*n* = 10) of rat was injected intraperitoneally at a concentration of 1 mg/kg/day for 10 days. After day 10, the grip strength of the four limbs of the rats was assessed using the electronic dynamometer (San liang Apparatus, Guangdong, China). Each rat underwent five tests with a rest of 5 min between each test. The median of the five tests was calculated as the limb grip strength of the rat.

The judgment of the sarcopenic model establishment is mainly confirmed by the limb grip strength and the sarcopenia index (SI). The rat models after modelling were considered to meet the criteria of sarcopenia when the limb grip strength [20] and SI [21] were significantly decreased in the DEX group compared with the CON group. The formula for calculating the SI value is as follows: SI = weight of the gastrocnemius muscle (mg) ÷ weight of the subject (g).

### MRI acquisition

Magnetic resonance scanning axial scanning of rat paraspinal muscles was performed using GE Discovery MR750W 3.0T magnetic resonance with a 16 channel longitudinally placed rat specific coil (CG-MUC49-H300-AG, Chenguang Medical Technologies, China). The rats were anesthetized with an intraperitoneal injection of pentobarbital sodium (40 mg/kg) and placed in a standard prone position. A towel was used to maintain their body temperature. The animal MRI scanning sequences included axial Multi-Echo iN Steady-state Acquisition (Mensa), axial DT-MRI, and sagittal T2WI. The specific scanning parameters were shown in Table 1.

**Table 1:** MRI scan sequence parameters of lumbar paravertebral muscles in rat of sarcopenia

Sequence	TR(ms)	TE(ms)	FOV	Matrix	Spacing	NEX		Slice Thickness	Echo Train Length
Mensa	36.4	6.6	8 × 8	130 × 130	0	/	1		/
T2WI	3610	120	10 × 10	130 × 130	0	2.0	1.2		20.0
DTI	7000	6.4	10 × 10	256 × 256	0	/	2.9		/

### MRI image analysis

**Fig. 1:**
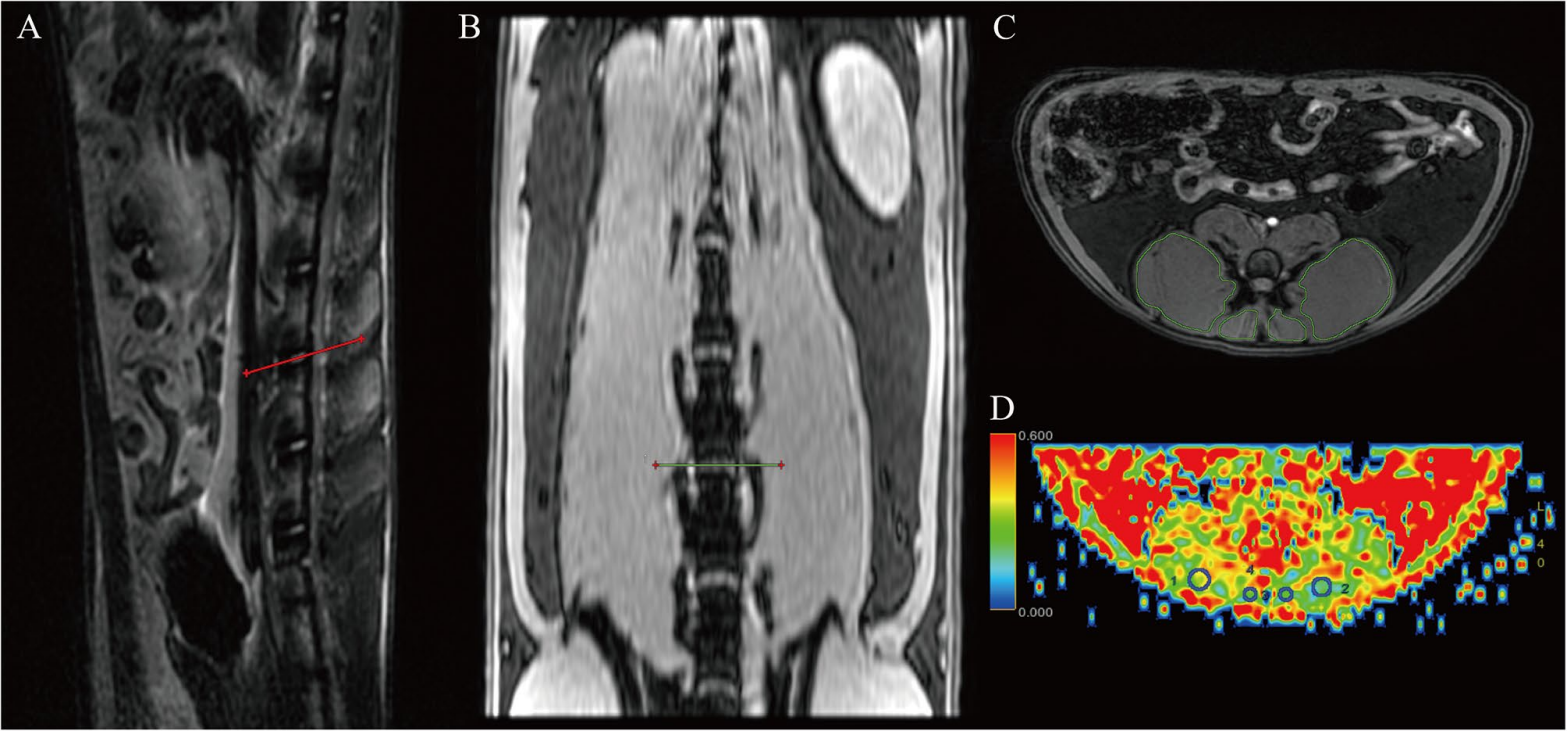
Schematic diagram of L4-5disc positioning and ROI delineation. (**A**) T2 sagittal localization map. (**B**) Mensa coronal localization map. (**C**) Cross-sectional Mensa schematic diagram for ROI delineation. (**D**) Coronal DTI schematic diagram for ROI delineation

The initial Mensa, T2WI, and DT-MRI images were sent for post-processing to the GE AW4.7 image workstation. By picking at the lumbar spine 4-5(L4-5) disc level in the sagittal images of Mensa serial [22], drawing the outline of the paraspinal muscle bilaterally along the intramuscular border and determining the maximum cross-sectional area (CSA) of the muscles. To reduce the partial volume effect of the muscle interface, circular regions of interest (ROI) with diameters of 3 mm and 1 mm were drawn at the center of the muscles for the erector spinae and multifidus, respectively, to measure DT-MRI-related parameters [23]. The ROI measurements for both the DEX and CON groups were taken three times at the same position, and the average value was calculated. The average value of the left and right target muscles is used as the final value for CSA and DT-MRI parameters. The measurement process of MRI is shown in Fig. 1.

### Tissue harvesting

Twenty-four hours after the last injection of DEX, the rats were weighed using an electronic scale and then euthanized by intraperitoneal injection of excessive pentobarbital sodium (40 mg/kg). The right gastrocnemius and erector spinae muscles were carefully dissected and excess fat and connective tissue were removed in saline. The gastrocnemius muscle was weighed. The proximal portion of the erector spinae was used for histological analysis, and the distal portion was used for molecular biological analysis. The samples of erector spinae for histological analysis were stored in 5 ml PE tubes filled with 4% paraformaldehyde. The samples of erecter spinae muscle samples for molecular biology analysis were snap-frozen in liquid nitrogen and then stored in a -80 °C freezer.

### Histological staining

The erector spinae muscle of the rat was fixed with 4% paraformaldehyde for 48 h, transferred to 20% sucrose overnight, and then embedded in paraffin blocks for sectioning. To evaluate the degree of changes in ECM in skeletal muscle, hematoxylin-eosin (HE) staining and sirius red staining were performed on longitudinal tissue sections with a thickness of 5 μm the erector spinae muscle layer. The collagen volume fraction (CVF) was calculated as the collagen content in the sirius red stained sections using ImageJ (CVF = total collagen area ÷ the total image area) [24].

### Western blot analysis

The erector spinae muscle was homogenized in RIPA lysis buffer containing protease inhibitor cocktail (RIPA Lysis Buffer System, sc-24,948; Santa Cruz Biotechnology, Dallas, TX, USA) and centrifuged at 10,000 g for 10 min at 4 °C, and supernatants were collected thereafter. The total protein concentration of supernatants was determined according to the Bradford method using the protein assay kit (Bio-Rad Laboratories, Hercules, CA, USA). Samples were diluted in 2 × Blue Loading Buffer and boiled at 95 °C for 5 min. Proteins (30 µg/lane) were loaded and separated on 8% or 12% sodium dodecyl sulfate-polyacrylamide gels. Proteins were blotted on the polyvinylidene fluoride membranes, and blocked for 1 h with 5% skimmed milk in PBS with Tween 20 (PBST). The membrane was incubated using antibodies against Collagen I (1:150 in PBST, AF7001; Affinity), Collagen III (1:150 in PBST, AF5457; Affinity), or Fibronectin (1:150 in PBST, AF5335; Affinity) overnight at 4 °C, and then incubated in a solution with horseradish peroxidase (HRP)-conjugated anti-mouse (1:12,000 in PBST) for 1 h. Proteins were detected using chemiluminescent reagents (Clarity™ Western ECL Substrate, Bio-Rad Laboratories, Hercules, CA, USA). Finally, images were analyzed with the LAS-1000 (Fujifilm, Tokyo, Japan) using a chemiluminescent image analyzer, and quantitative analysis of protein expression of collagen I, collagen III, and fibronectin were performed using ImageJ software (version 1.52a, Wayne Rasband National Institutes of Health, Bethesda, Maryland, USA).

### Statistical analysis

Statistical analysis was conducted using SPSS version 26.0 (IBM Corp, Armonk, NY, USA) and GraphPad Prism 8.0 (GraphPad Software, La Jolla, CA, USA). We used the bilateral significance test to determine differences between groups, with two- sided *p <* 0.05 considered to be statistically significant. Variables with a normal distribution are expressed as mean ± standard deviation (SD) and the variables with a skewed distribution are expressed as median (interquartile range). Two independent sample *t-*tests were used for variables with normal distribution, while Mann-Whitney U tests were used for skewed distribution. Correlation coefficients were calculated to test the strength of associations among collagen I, collagen III, fibronectin, CVF, limb grip strength and FA value using the Pearson method.

## Result

### Modeling Assessment

**Fig. 2:**
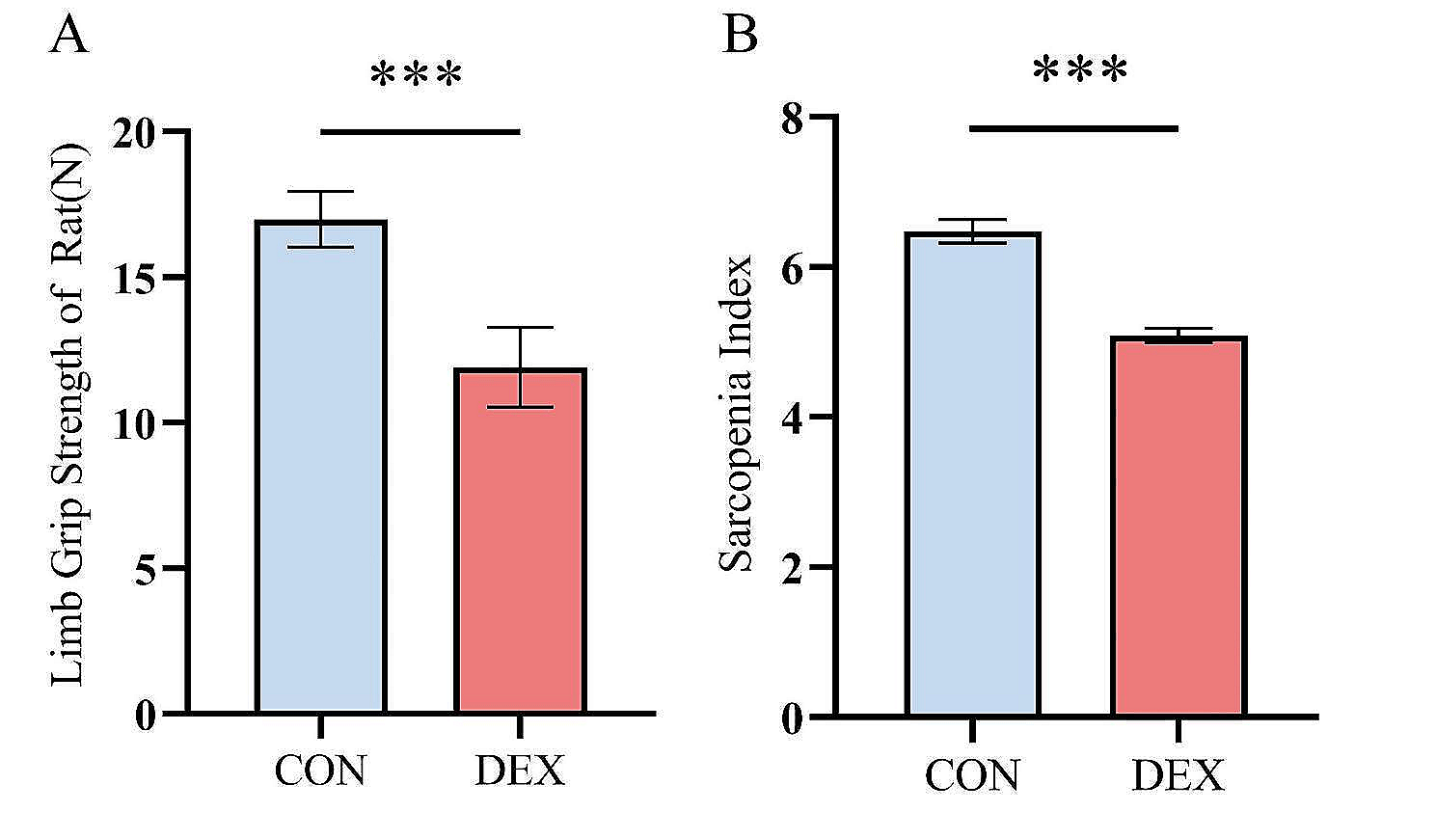
Statistics of limb grip strength and SI results in the CON versus DEX groups. CON = control. DEX = dexamethasone. ***p < 0.001

This study was conducted to verify whether the rat models after modelling meet the criteria of sarcopenia by evaluating the grip strength of the four limbs and the SI value. The results of the modeling evaluation are presented in Fig. 2. The grip strength of all four limbs was significantly reduced in the DEX group compared to the CON group (16.99 ± 0.95 vs. 11.91 ± 1.37, *p* < 0.001). The SI value of rats was significantly reduced in the DEX group compared to the CON group (6.45 ± 0.10 vs. 5.07 ± 0.07, *p* < 0.001). From the foregoing, it was demonstrated that the rat model of sarcopenia was successfully constructed.

### Paralumbar muscles MRI analysis

**Fig. 3:**
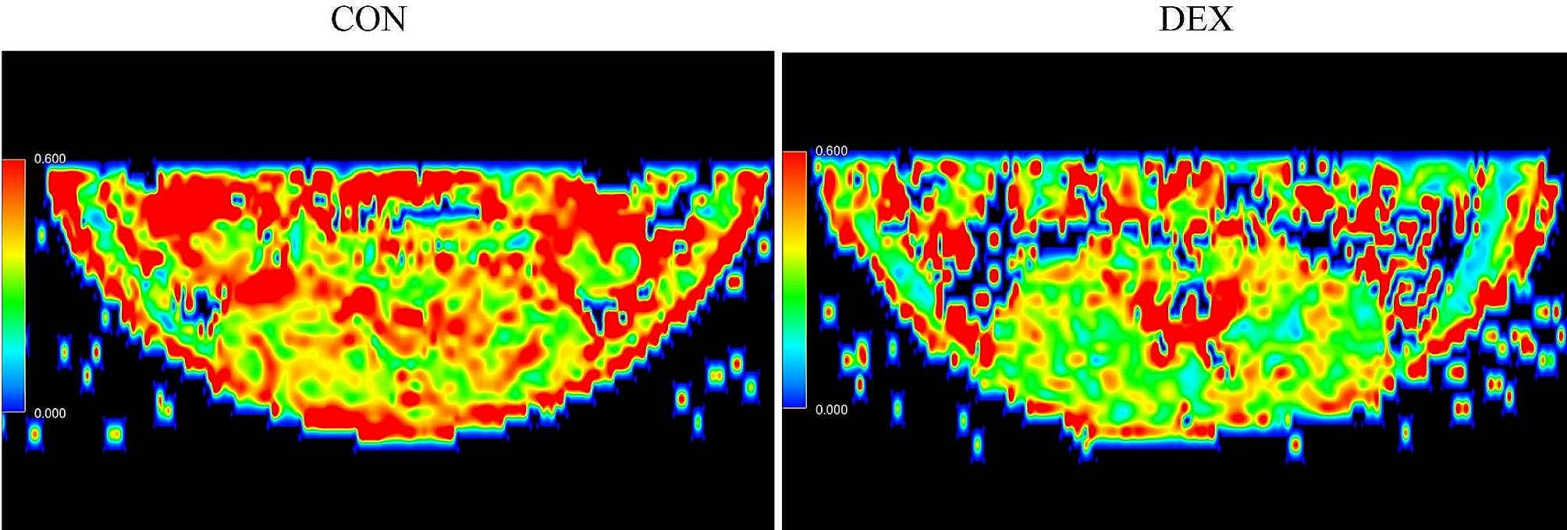
Comparison of representative pseudo-colour DTI-FA images of intervertebral discs at the L4-5 level in the CON and DEX groups. CON = control. DEX = dexamethasone

The radiographic results indicate that the fractional anisotropy (FA) values of the erector spinae in the DEX group were significantly lower than those in the CON group (*p* < 0.01). The mean diffusivity (MD) values of the erector spinae in the DEX group were higher than those in the CON group, but there was no statistical difference. The maximum CSA of the erector spinae in the DEX group was significantly reduced compared to that in the CON group with statistical differences (*p* < 0.01). The FA values of the multifidus muscle of rats in the DEX group were lower than those in the CON group, but there was no statistical difference. The maximum CSA of the multifidus muscle in the DEX group was lower than that in the CON group, although there was no statistical difference (*p* > 0.05). The MD value of the multifidus muscle in the DEX group was higher than that in the CON group, but there was no statistical difference. Figure 3 shows the DTI-FA pseudo-colour image of the lumbar intervertebral disc at the L4-L5 disc level. Specific values for each imaging parameter are detailed in Table 2.

**Table 2:** CSA, FA, MD values (Mean ± SD) of erector spinae and multifidus at L4 / L5 disc level in CON group versus DEX group. *N* = 10. CSA: cross-sectional area. FA: fractional anisotropy. MD: mean diffusion. CON = control. DEX = dexamethasone

Sequence	Erector spinae			Multifidus			
	CON	DEX	*p* value	CON	DEX	*p* value	
CSA (mm^2^)	89.62 ± 3.82	74.77 ± 6.74	< 0.001	6.23 ± 0.20	6.44 ± 0.32		0.09
FA (-)	0.33 ± 0.02	0.29 ± 0.02	< 0.001	0.33 ± 0.01	0.32 ± 0.01		0.11
MD (µm^2^/s)	1.34 ± 0.12	1.47 ± 0.08	0.52	1.32 ± 0.08	1.34 ± 0.12		0.65

### Histological staining results

**Fig. 4:**
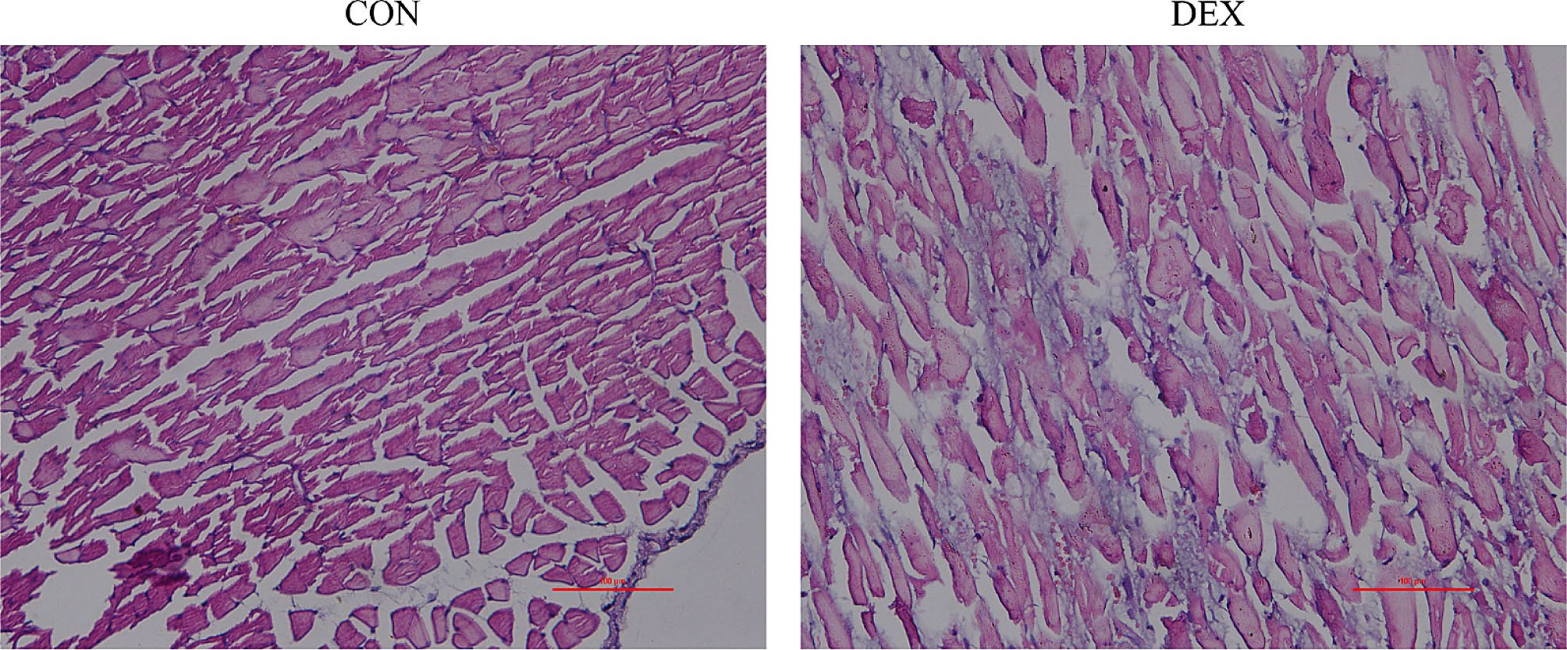
Representative HE staining of the erector spinae muscle in CON group versus DEX group. Scale bar, 100 μm. CON = control. DEX = dexamethasone

**Fig. 5:**
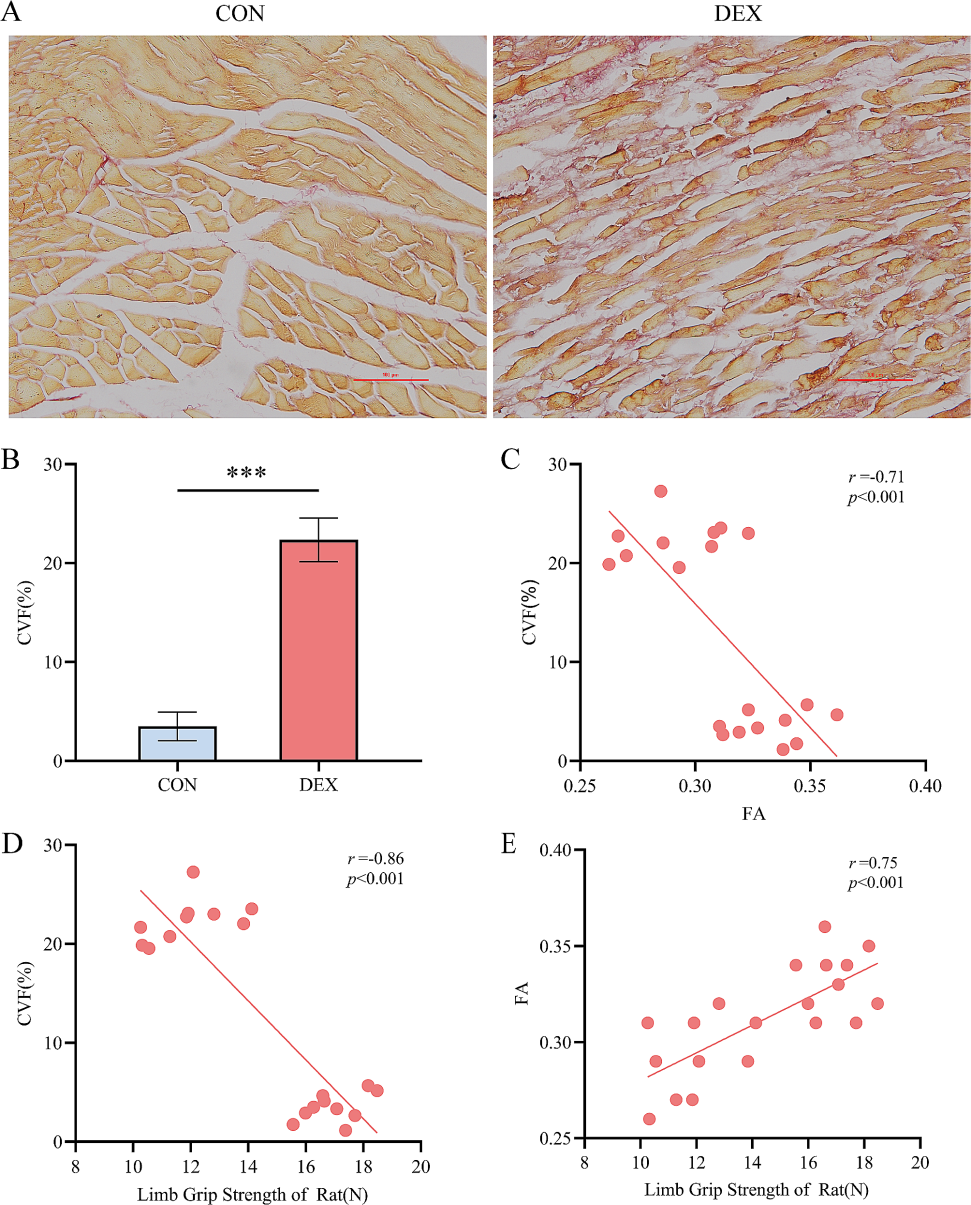
(**A**) Representative sirius red staining of the erector spinal muscle in CON group versus DEX group. (**B**) Sirius red staining quantitative analysis histogram. C-E. Correlation scatter plot. *N* = 10. Scale bar, 100 μm. *** *p* < 0.001. CON = control. DEX = dexamethasone. CVF = collagen volume fraction

HE staining in Fig. 4 showed that the muscle fibers in the CON group were regularly arranged, with uniform intermuscular spaces and no inflammatory infiltration. In contrast, the DEX group exhibited distorted and disorganized myofibers, a decrease in their number, and enlarged extracellular spaces with inflammatory infiltrates and fiber proliferation. Sirius red staining is a classic connective tissue staining method that can stain collagen fibers in ECM as pink and skeletal muscle cells as yellow. Figure 5 shows that collagen fibre deposition was significantly higher in the DEX group compared to the CON group, as demonstrated by sirius red staining. The CVF was also significantly higher in the DEX group than in the CON group (*p* < 0.001).

### Western blotting analysis

**Fig. 6:**
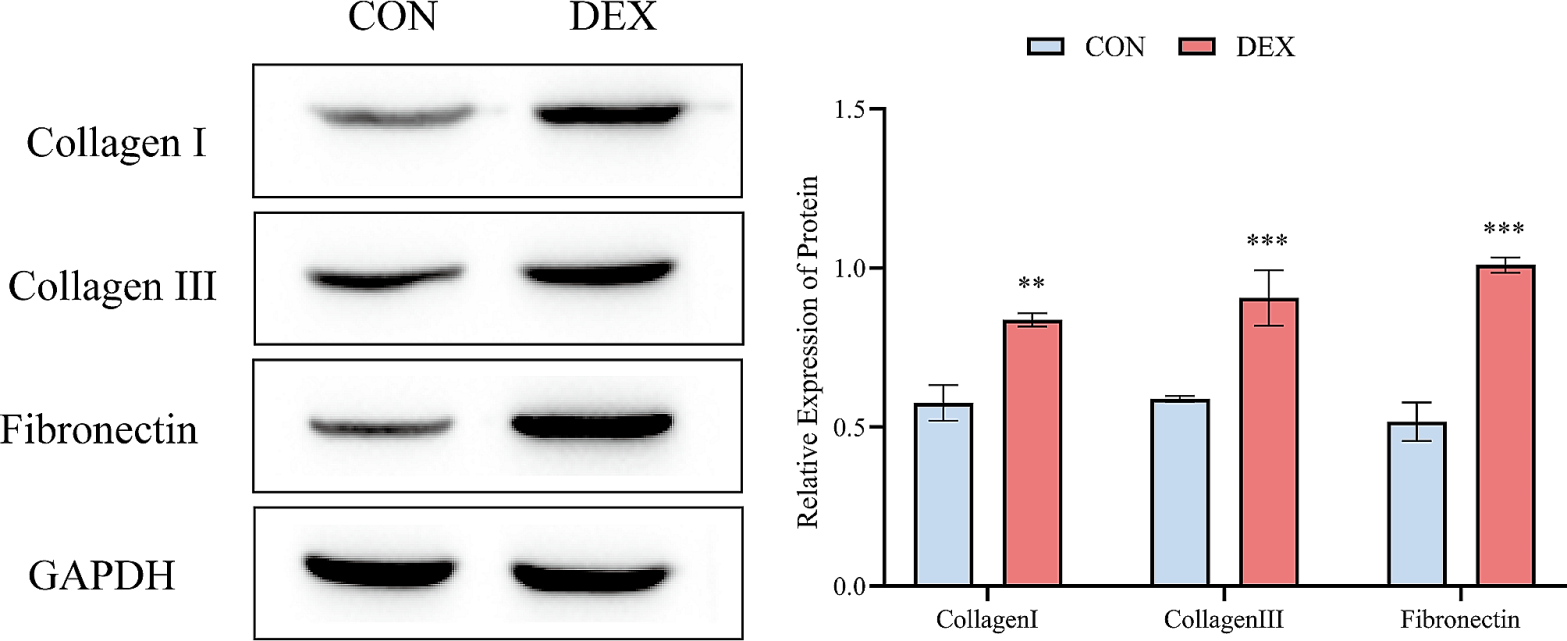
Quantitative analysis of Collagen I, Collagen III, and Fibronectin in CON and DEX groups by western blot analysis. Glyceraldehyde-3-phosphate dehydrogenase (GAPDH) was used as a loading control. ** *p* < 0.01, *** *p* < 0.001. CON = control. DEX = dexamethasone

The collagen fibers in skeletal muscle mainly contain type I and type III, therefore we performed western blotting on collagen fiber-related proteins to detect their protein deposition. Figure 6 shows that, compared to the CON group, the relative expression of protein (relative expression of protein = target protein gray scale values ÷ internal reference protein gray scale values) of type I, type III collagen and fibronectin in the DEX group were significantly increased. According to the Pearson correlation analysis, the results of the correlation analysis between FA and parameters such as CVF, collagen I, collagen III and fibronectin are shown in Table 3. The analysis revealed strong correlations between FA values and pathological CVF, collagen I, collagen III, and fibronectin.

**Table 3:** Correlation analysis between FA values with CVF, collagen I, collagen III, and fibronectin

	CVF	*p* value	Collagen I	*p* value	Collagen III	*p* value		Fibronectin	*p* value
FA	-0.71	< 0.001	-0.94	< 0.01	-0.85	0.03	-0.88		0.02

## Discussion

Our results indicate that the FA value of the erector spinalis muscle in rats from the DEX group was significantly reduced compared to the CON group. This finding is consistent with the significant difference in ECM connective tissue content shown by sirius red staining and is highly correlated. The FA value can be used as a non-invasive imaging biomarker to evaluate the remodeling of the ECM of skeletal muscle. It reflects, to some extent, the collagen deposition and ECM dysfunction of erector spinae in sarcopenic rats. This information may provide valuable for monitoring disease progression and evaluating prognosis.

Muscle mass is a term used to describe the structure and composition of muscles at both macroscopic and microscopic levels [25]. At the macro level, it mainly refers to changes in muscle size and number. In our study, the CSA value of the erector spinalis muscle was significantly reduced in the DEX group rats compared to the CON group, while the CSA value of the multifidus muscle did not show significant changes. From the perspective of muscle fiber types, this may be due to the fact that the erector spinalis muscle is primarily composed of type II muscle fibers, while the multifidus muscle is primarily composed of type I muscle fibers [26]. At the micro level, this mainly refers to the changes in muscle quality related factors that maintain muscle quantity, including muscle composition, muscle fibrosis, blood flow perfusion, and fat infiltration, etc. Collagen is a major structural protein in the ECM, and the major components include collagen types I versus III [9]. Collagen I can resist tensile stress, generating tension and stiffness in skeletal muscles. On the other hand, Collagen III is more elastic, ensuring the compliance of skeletal muscles [10]. Muscle fibrosis is a significant pathological change that occurs during the aging process of skeletal muscles [8]. The deposition of collagen and fibrosis in skeletal muscle is a characteristic pathological change of ECM remodeling [7]. The sirius red staining results indicate that the DEX group of rats showed a significant increase in interstitial fibrous connective tissue in skeletal muscle compared to the CON group. Furthermore, Hindle et al. reported that the process of ECM remodeling, which is associated with aging, is accompanied by changes in the biochemical composition of the ECM. These changes are characterized by a higher proportion of type I and III collagen fibers in the elderly [27]. This is consistent with our research findings, where the content of collagen I and collagen III in the erector spinae muscle of rats in the DEX group was significantly higher than that in the CON group. Mahdy et al.‘s research [11] found that the accumulation of fibrous tissue increases the stiffness of skeletal muscles, which limits their extension and contraction, ultimately reducing athletic ability. This is consistent with our research findings, where there is a strong correlation between rat limb grip strength and ECM remodeling related indicators.

DT-MRI is a technology that is sensitive to the random movement or diffusion of water molecules in tissues. It can non-invasively detect the ultrastructure and pathological changes of organisms at the molecular level [28]. The application of DT-MRI in the central nervous system has become very mature [29] and is increasingly being used in the musculoskeletal system to detect and quantify pathological and physiological changes, such as skeletal muscle fibrosis [30]. Our DT-MRI quantitative analysis results showed that the FA values of the erector spinae in the DEX group were significantly decreased compared to the CON group. However, the FA values of the multifidus in the DEX group did not differ significantly from those in the CON group, possibly because DEX had little effect on multifidus muscle injury, which was mainly composed of type I muscle fibers. The FA represents the percentage of the anisotropic component of water molecules in the entire diffusion tensor. The magnitude of the FA value, which represents the integrity and directionality of the cell membrane and muscle fibers, was used to quantify the orientation of water molecules as an indirect indicator for evaluating microstructural fiber changes. The smaller the FA value, the worse the anisotropy of water molecule diffusion, indicating that the arrangement of tissue fibers is more disordered and discontinuous [31]. The range of FA values is between 0 and 1. Our results suggest that the decrease in FA value may be due to increased collagen deposition in aging muscle tissue induced by DEX, which limits the movement of water molecules within the tissue. A human DT-MRI study in Farrow [14] demonstrated the ageing of skeletal muscle with increasing age, with lower FA values and higher MD values compared to younger individuals. This was consistent with the trend of our imaging results. In addition, in our analysis of the correlation between imaging and pathology, we found that FA values were strongly correlated with CVF. MD is the average of the sum of the diffusion tensors in each direction and mainly reflects the speed of diffusion movement. The application of DT-MRI related parameters as non-invasive imaging markers for monitoring ECM remodeling in muscle may provide valuable information for further early diagnostic studies of sarcopenia. In addition, due to the lack of effective drugs for the treatment of mid to late stage sarcopenia [32], DT-MRI technology can provide non-invasive monitoring and dynamic evaluation of the efficacy of new drugs for skeletal muscle fibrosis. Compared to invasive biopsy sampling, DT-MRI technology has the advantages of zero trauma, reproducibility, and multiple detections. The pathological results of this study further confirm that the DEX-induced sarcopenia rat model can not only induce skeletal muscle atrophy dominated by type II muscle fibers, but also lead to collagen fiber deposition.

Sarcopenia is a systemic skeletal muscle disease. However, previous research has mainly focused on lower limb muscle groups, such as the quadriceps femoris, gastrocnemius, and soleus, with relatively little MRI evaluation of the lumbar paravertebral muscle groups. The lumbar paravertebral muscles, as the core muscle group in the body, play a vital role in stabilizing the spine versus pelvis [33]. The structures and components of the lumbar paravertebral muscles are gaining increasing attention as potential prognostic and diagnostic markers of spinal health [34]. In addition to maintenance of spine health, psoas muscle mass has been used to predict mortality in cirrhotic [35], colorectal surgery [36], post left ventricular assist device implantation [37], and results after disc surgery [38]. On the other hand, with increasing attention being paid to value-added opportunistic imaging [39], the use of existing abdominal imaging to provide additional body composition analysis further enhances its importance and value in clinical practice.

Natural aging rats are a good model for studying sarcopenia. However, constructing such a model has limitations, such as a long feeding cycle and high cost. An alternative model of sarcopenia induced by DEX has been increasingly used by scholars [40]. This model can induce an aging model of sarcopenia in a short period of time. DEX mainly causes type II muscle fiber atrophy [41],which is consistent with the pattern of skeletal muscle aging in the human body. In terms of animal research, Wang et al. compared DEX-induced aging rats with naturally aged rats and found high consistency between the two models [20]. During clinical intervention, patients with congenital adrenal hyperplasia [42] and Addison’s disease [43] may require lifelong DEX treatment, which increases the risk of sarcopenia. Criteria for judging the successful establishment of the model mainly refer to the diagnostic criteria for human sarcopenia: significant reduction in muscle strength and mass [44]. The criteria for evaluating the successful establishment of a rat model of sarcopenia primarily refer to the clinical diagnostic criteria of sarcopenia [45]: significant reduction in muscle strength and mass. In our results, the grip strength of four limbs and SI value were significantly decreased in the DEX group compared to the CON group, indicating the successful establishment of a rat model with sarcopenia.

There are some limitations to this study. Firstly, we did not evaluate other magnetic resonance sequences, such as the IDEAL-IQ, which may provide additional information on muscle fatty infiltration [46]. Secondly, there is species heterogeneity between humans and animals. As this is an animal study, the subject of the study is a closed group of Sprague-Dawley rats. Consequently, the impact of genetic and environmental factors on the results is relatively limited. In future clinical research validation, it is necessary to consider a greater number of potential confounding factors, such as age, gender, race, etc. Finally, due to the small sample size, we did not dynamically evaluate animal models at different time periods. However, we conducted an in-depth histopathological validation. In the future, we will conduct further longitudinal clinical studies to evaluate the early evaluation value of DT-MRI imaging markers in sarcopenia.

## Conclusions

In conclusion, his study establishes that DT-MRI parameter FA values can serve as a potential non-invasive imaging marker for monitoring ECM remodeling in skeletal muscle. The FA value strongly correlates with pathological CVF, collagen I, collagen III, and fibronectin. These results provide potential clinical tools for assessing the pathophysiological changes of ECM remodeling in skeletal muscle and effective imaging biomarkers for further early diagnostic studies of sarcopenia.

### Electronic supplementary material

Below is the link to the electronic supplementary material.

**Supplementary Material 1:** The full uncropped Gels and Blots images of Collagen I

**Supplementary Material 2:** The full uncropped Gels and Blots images of Collagen III

**Supplementary Material 3:** The full uncropped Gels and Blots images of Fibronectin

**Supplementary Material 4:** The full uncropped Gels and Blots images of GAPDH

## Data Availability

No datasets were generated or analysed during the current study.

## Declarations

### Statement

The study is reported in accordance with ARRIVE guidelines (https://arriveguidelines.org).

## Abbreviations

ECM: Extracellular matrix
MRI: Magnetic resonance imaging
DT-MRI: Diffusion tensor magnetic resonance imaging
DEX: Dexamethasone
CON: Control
SI: Sarcopenia index
Mensa: Multi-echo iN steady-state acquisition
CSA: Cross-sectional area
ROI: Regions of interest
HE: Hematoxylin-eosin
CVF: Collagen volume fraction
SD: Standard deviation
HRP: Horseradish peroxidase
FA: Fractional anisotropy
MD: Mean diffusivity
GAPDH: Glyceraldehyde-3-phosphate dehydrogenase
