# Supplementary figures and images for: Diffusion-tensor magnetic resonance imaging as a non-invasive assessment of extracellular matrix remodeling in lumbar paravertebral muscles of rats with sarcopenia
Source: BMC Musculoskelet Disord. 2024 Jul 13;25:540. doi: 10.1186/s12891-024-07654-0 (PMC11245810; doi:10.1186/s12891-024-07654-0)

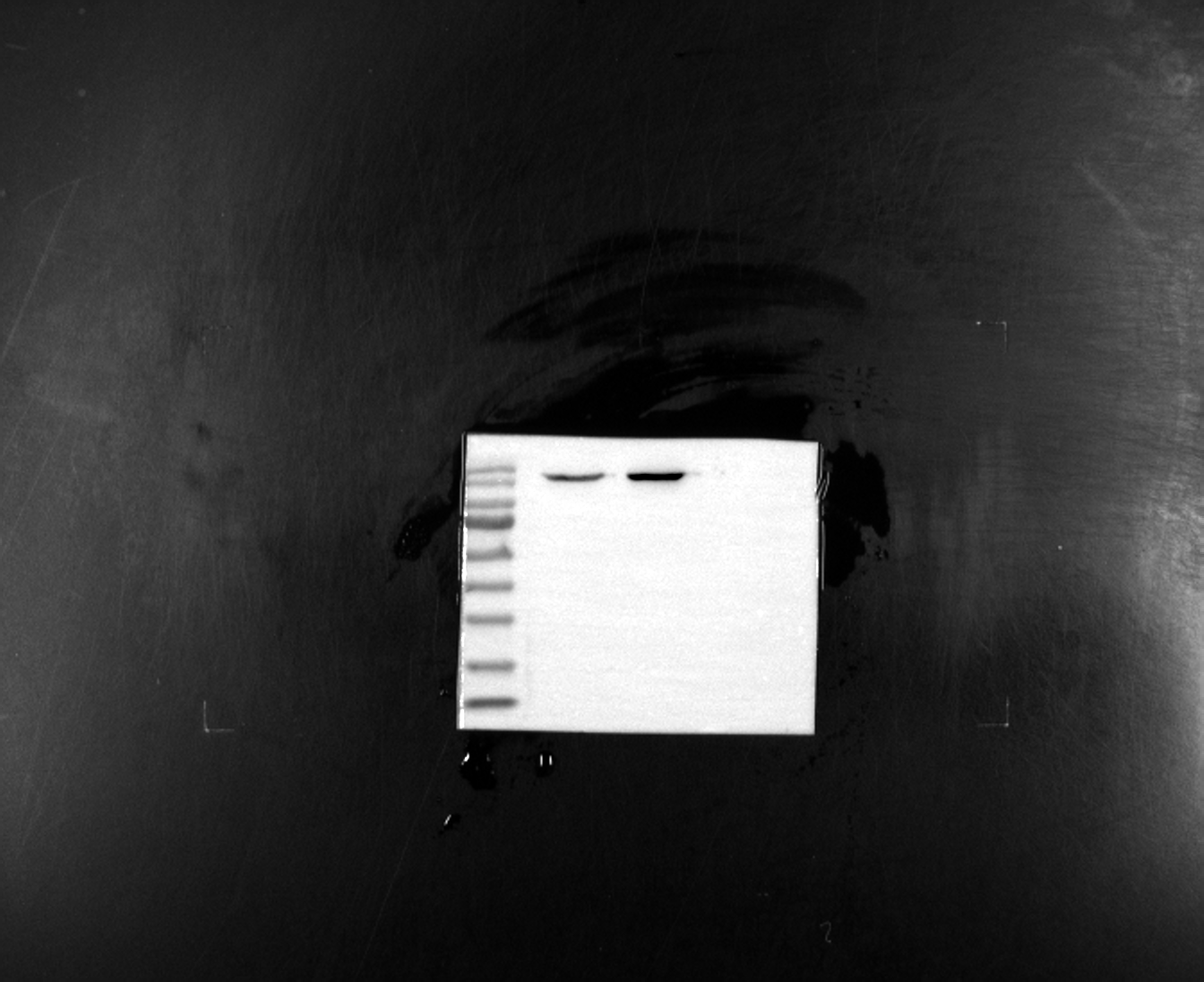

Supplement: Supplementary file 1 — Supplementary Material 1: The full uncropped Gels and Blots images of Collagen I [file 12891_2024_7654_MOESM1_ESM.tif]

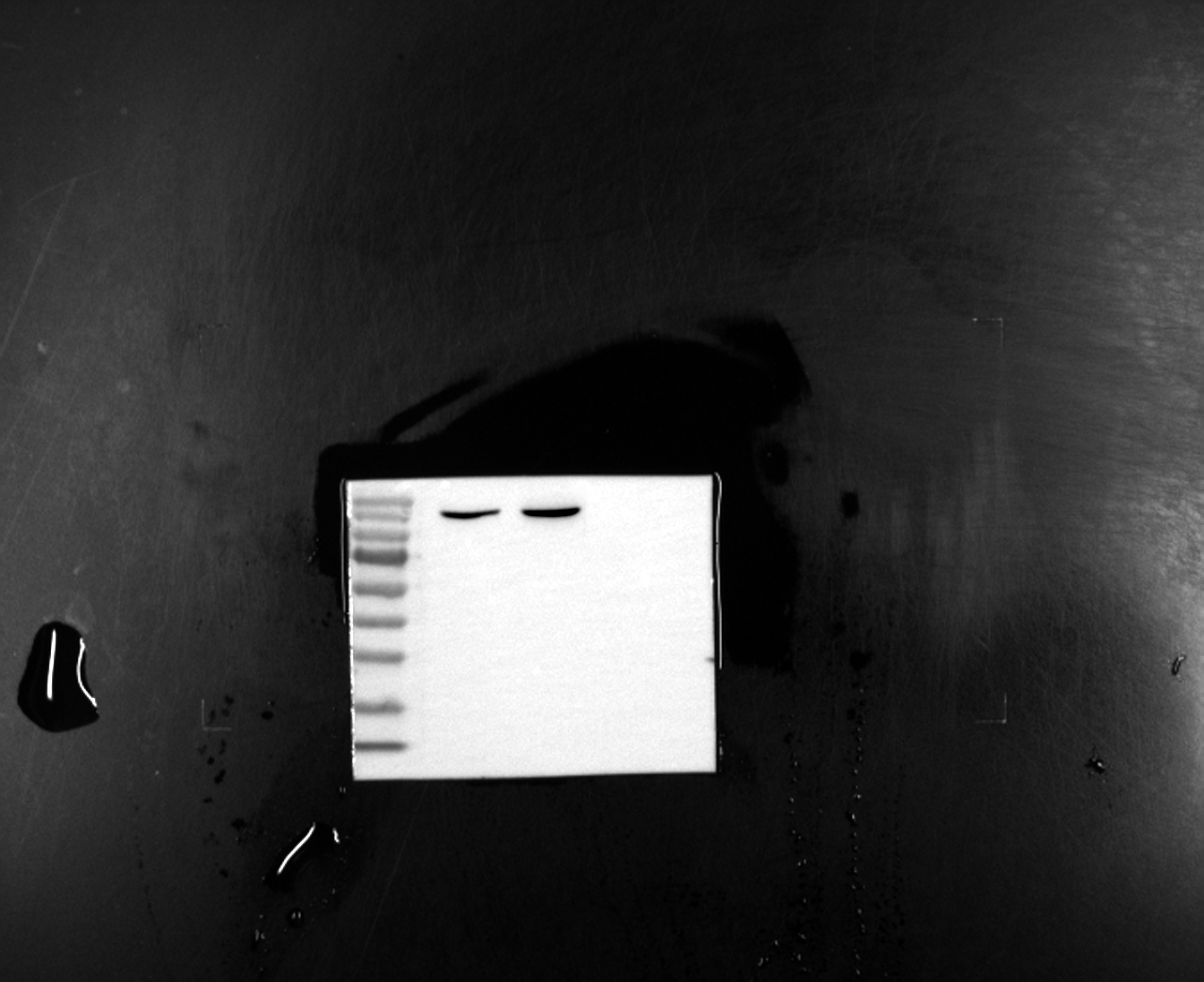

Supplement: Supplementary file 2 — Supplementary Material 2: The full uncropped Gels and Blots images of Collagen III [file 12891_2024_7654_MOESM2_ESM.tif]

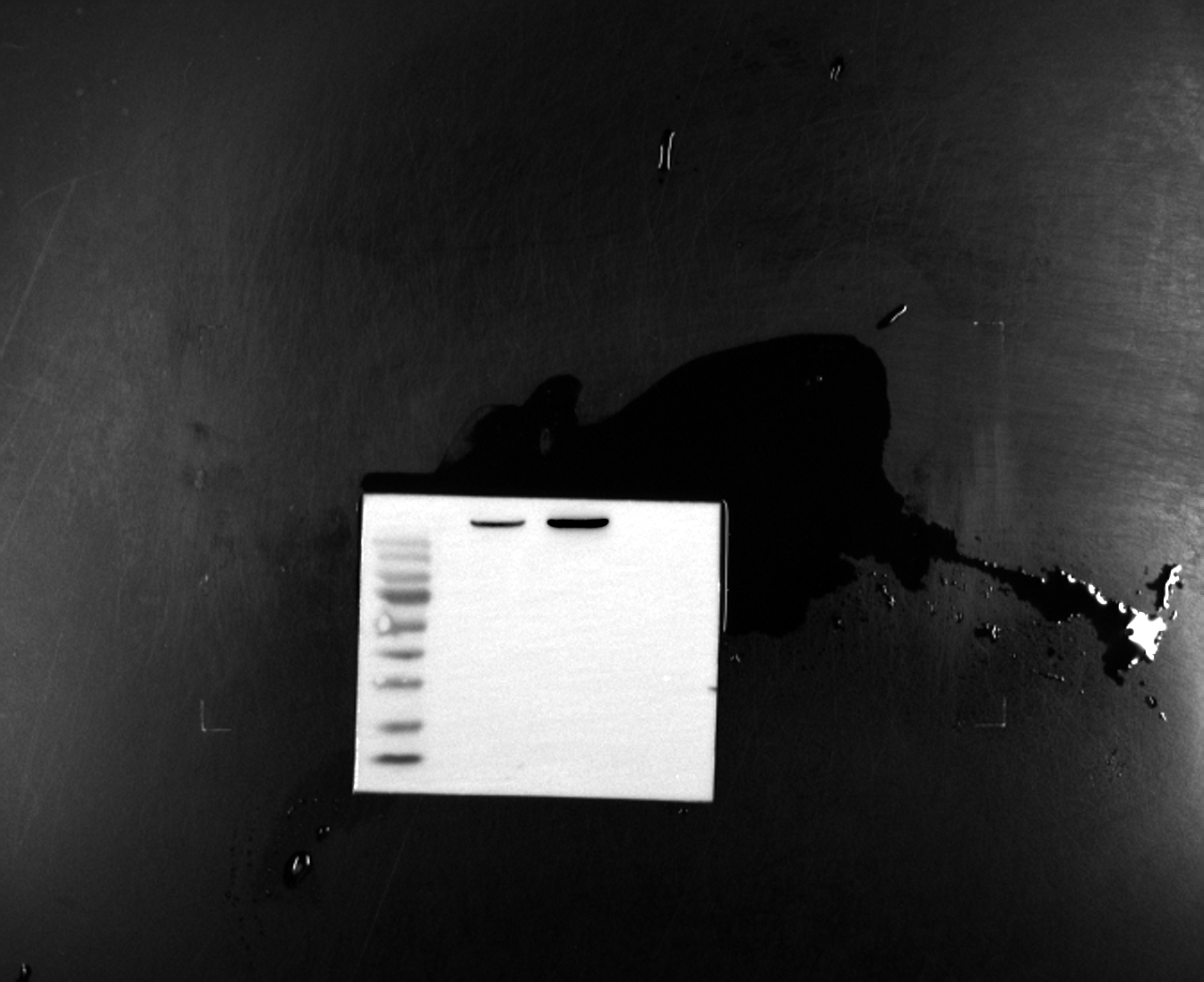

Supplement: Supplementary file 3 — Supplementary Material 3: The full uncropped Gels and Blots images of Fibronectin [file 12891_2024_7654_MOESM3_ESM.tif]

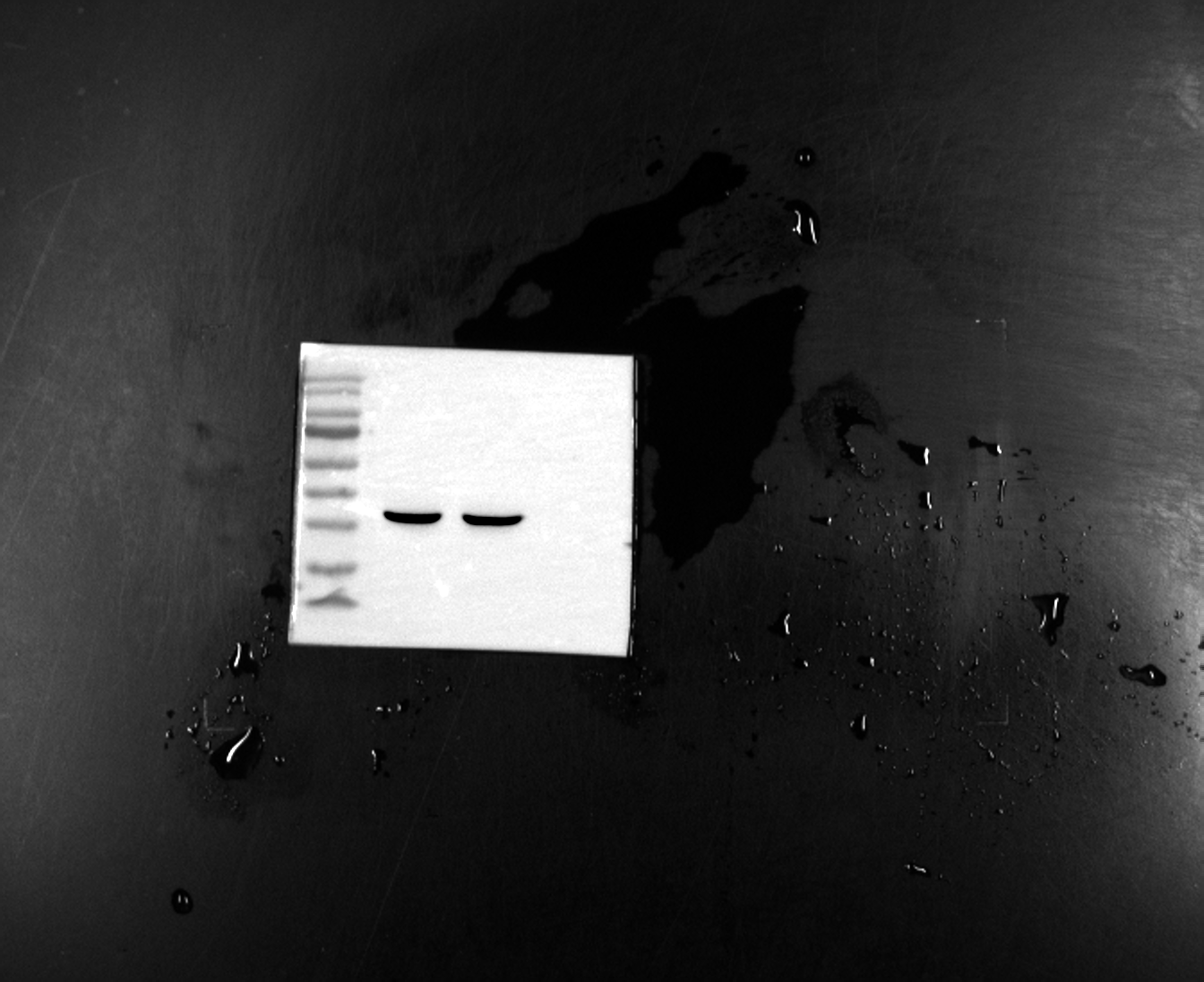

Supplement: Supplementary file 4 — Supplementary Material 4: The full uncropped Gels and Blots images of GAPDH [file 12891_2024_7654_MOESM4_ESM.tif]
